# Radiomics in precision oncology: hype or *ludum mutante*

**DOI:** 10.1186/s12916-023-03165-2

**Published:** 2023-11-28

**Authors:** Shi Hui Tay, Xin Zhang, Melvin L. K. Chua

**Affiliations:** 1 Precision Radiotherapeutics Oncology Programme, Division of Medical Sciences, National Cancer Centre Singapore, 30 Hospital Boulevard, Singapore, 168583 Singapore; 2 Radiation Oncology Center, Chongqing University Cancer Hospital, Chongqing, China; 3 Department of Head and Neck and Thoracic Radiation Oncology, National Cancer Centre Singapore, 30 Hospital Boulevard, Singapore, 168583 Singapore; 4 Oncology Academic Clinical Programme, Duke-NUS Medical School, Singapore, Singapore

**Keywords:** Radiomics, Imaging, Precision oncology, Explainable AI, Nasopharyngeal cancer

## Background

Precision oncology has emerged as the next frontier of therapeutic strategies in the treatment of human cancers. The idea is simple yet appealing: to decipher the genotypes and phenotypes of cancer, so that risk prediction and therapies can be individualised for every patient. To this end, next-generation sequencing (NGS) tools have been developed to characterise the genomics of tumours. While there have been successes with this approach in terms of matching effective therapies to genotypes, testing is however restricted by the availability of tumour tissue. Consequently, research has explored using imaging as a data source for deeper phenotyping, given that radiological scans, e.g. computed tomography and magnetic resonance imaging (MRI), are routinely performed for cancer diagnosis, assessment of treatment response, and disease surveillance. Radiomics thus represents a domain of science that involves extraction of quantitative imaging features pertaining to shapes, grey-level textures, and intensities that are non-perceivable to the human eye from specific regions of interest (ROI) delineated on radiological images.

### Radiomics tools for cancer diagnosis and clinical stratification

Over the years, several studies have reported on the promise of radiomics for diagnosis and clinical stratification of phenotypes for treatment intensification or de-intensification [1]. These published radiomics models purport to prognosticate outcomes of cancer patients and/or predict their response to a specific drug [2, 3]. Nonetheless, the implementation of radiomics in the clinic remains challenging, partly due to poor model reproducibility. The Image Biomarker Standardisation Initiative (IBSI) was thus launched to standardise the radiomics workflow [4]. Separately, the radiomics quality score (RQS) was introduced to assist clinicians with evaluating the quality of radiomics studies [5].

It is in this background that we appraise the study by Liu and colleagues[6] who developed a radiomics signature to predict post-radiation nasopharyngeal necrosis (PRNN) in patients with locoregionally-recurrent nasopharyngeal carcinoma (lrNPC). For model development, the investigators utilised pre-treatment MRI images (T1-weighted with and without contrast-enhancement, and T2-weighted sequences) of 761 patients (split into 420 for training, and 341 for validation) enrolled from four hospitals. Using a random forest model, they built a 6-feature signature consisting of 1 first-order statistic, 2 shape features, and 3 texture features that could discretise patients into low- and high-risk for PRNN. The signature achieved AUCs of 0.722 in the training dataset, and 0.713 and 0.756 for the internal and external validation cohorts, respectively. The signature outperformed known prognostic clinical predictors, including gross tumour volume, age, disease-free interval, sex, and re-irradiation dose [7]. It was also generalised across different centres, imaging parameters, and patient subgroups (e.g., different ages and rT-categories), with AUCs ranging from 0.671 to 0.888. To provide explanability for the model, the investigators correlated the radiomics features with somatic transcriptomic profiles of 29 patients. From gene set enrichment analyses, they associated the 6 radiomics features with fibrosis and vascularity signalling pathways.

### Strengths and limitations

Overall, there are several strengths of this study. First, PRNN is an important and clinically relevant outcome in lrNPC patients who are being planned for re-irradiation; for these patients, soft tissue necrosis following re-irradiation is a common and potentially debilitating toxicity [8]. Thus, having a tool that can assist with patient selection is advantageous from the clinical perspective. Second, the study investigators asserted the reliability of their radiomics model by undertaking comprehensive steps for validation, which included proving its generalisability across different disease states and institutions.

That said, do we anticipate the deployment of this radiomics tool in the clinic tomorrow? Some notable limitations deserve mention. First, the AUCs of ~ 0.7 for prediction are modest at best. Second, it is uncertain if the samples used for the transcriptomic profiling were spatially correlated with the ROI from which the radiomics features were extracted. This is a crucial consideration when interpreting the robustness of the radio-transcriptomic analyses. Third, the lack of comprehensive documentation, provision of open-source codes, and data availability pose substantial challenges to assess model reproducibility.

### Translation of radiomics tools from research to clinic

Ultimately, what are the radical steps needed to bridge the deployment of radiomics tools from research to the clinic?

1. Standardisation of radiomics workflow: Harmonising the processes from image acquisition to model validation is a key step. To promote adherence, the IBSI working group had derived a set of guidelines for benchmarking of future radiomics studies [4].
2. Automation of ROI segmentation: This step is important since radiomics feature extraction is highly sensitive to subtle variations in segmentation methods [9].
3. Ensuring data quality: For external validation of radiomics models, we propose the need for benchmarking criteria to appraise the quality of datasets relating to the accuracy of clinical annotation and the extent of data missingness, given that these parameters can skew model performance.
4. Transparency of study results and validation: Instead of solely relying on the investigators, efforts must be made to encourage validation studies by independent groups within a defined window period. The results of these studies should be made transparent regardless of their outcomes (positive or negative validation), and journals should commit to publishing them. To achieve this, detailed reports, source codes, and anonymised data from the original study must be made available.
5. Explanability of the radiomics model: We surmise it would be best practice for radiomics models to include clinical and biological associations that underpin their development. This could be achieved by either spatially correlating the radiomics indices to molecular profiles or treatment response within the ROI [10].
6. Spatial-level resolution of radiomics features: Distinct regions within a ROI may exhibit differential treatment responses. Thus, interrogating spatial-level radiomics may enhance its explanability compared with bulk-level radiomics, and represents an exciting direction for the field.

## Conclusions

The oncology community remains in *limbo* about the relevance of radiomics in precision oncology, even though studies continue to report on its promise. Looking ahead, there needs to be a pivot in focus from reporting another “*hyped*” radiomics model to showcasing scientific robustness for clinical implementation. This would entail adopting some of our proposed measures and to eventually test these models in prospective radiomics-directed clinical trials. Only then, will radiomics fulfil its promise as a “*ludum mutante*” in precision oncology.

## Data Availability

Not applicable.
